# Anorectal Malformations: Histomorphological and Immunohistochemical Evaluation of Neuronal Dysfunction

**DOI:** 10.21699/jns.v6i2.559

**Published:** 2017-04-15

**Authors:** Yashika Bhatia, Sunita Singh, Kamal Nain Rattan, Padam Parmar, Divya Sahni, Rajeev Sen

**Affiliations:** 1 Department of Pathology, PT BDS PGIMS, Rohtak, Haryana; 2 Department of Pediatric Surgery, PT BDS PGIMS, Rohtak, Haryana; 3 Department of Medicine, PT BDS PGIMS, Rohtak, Haryana

**Keywords:** Anorectal malformations, ICCs, CD117, Calretinin, Biopsy.

## Abstract

**Objective:**

The patients with anorectal malformations (ARM) have been identified with specific and non-specific pathological changes. The present study was conducted with the aim to study histomorphological changes and various immunohistochemical (IHC) markers (calretinin, S-100, CD117) in intestinal wall specimens to assess neuronal dysfunction in ARM patients.

**Material and methods:**

Thirty children having ARM were included in our study. In all the cases, a representative biopsy was received. The tissue sections were processed and wax blocks were prepared. Various histopathological changes were examined on routine H&E. Representative sections were further subjected to IHC staining for ganglion cells (calretinin), interstitial cells of Cajal (CD117) and nerve bundles (S-100 protein). Descriptive variables were analyzed to assess neuronal dysfunction in cases of ARM. Chi-square was used to compare the categorical values. P-value <0.05 was accepted as statistically significant.

**Results:**

Biopsies were studied for histological changes using H&E stain. The most frequently observed histological finding in mucosa was inflammation and congestion in 87% and 67% of cases respectively. Disrupted muscularis mucosa was observed in 60%, eroded mucosa in 57%, and hemorrhage in 40% of cases. Submucosal inflammation and congestion were most common finding observed in submucosa in 87% and 80% cases respectively. CD117 was used to demonstrate altered density and distribution of interstitial cells of Cajal (ICC) in cases of ARM. Majority of them belong to grade 2+ category (n=17, 57%) followed by grade 1+ (n=8, 17%) for ICC cells. Altered density and distribution of ICC was observed in ARM which was statistically significant (p=0.02).

**Conclusion:**

The malformed segments in ARM show various specific and non-specific histomorphological changes. Examination of H&E sections along with IHC stains evaluation can minimize need for repeated biopsies and unnecessary radical treatment. CD117 immunohistochemistry is reliable adjunctive test in evaluation of ICC in motility disorders of bowel. Calretinin is good marker for identification of ganglion cells. In ARM, density and distribution of ICCs is significantly altered which can explain postoperative dysmotility.

## Introduction

Enteric nervous system (ENS), smooth muscle layer and interstitial cells of Cajal (ICC) are important for normal functioning of gastrointestinal tract.[1] Any defect involving these systems can result in variety of conditions like intestinal atresia, stenosis, malrotation, volvulus, meconium ileus, Hirschsprung disease (HD), colonic atresia, anal atresia and anorectal malformations (ARM) and present as neonatal bowel obstruction.[2] The need to demonstrate the various neuronal and myopathic changes in the bowel of patients with ARM using histopathological examination with H&E stain and ancillary tests like IHC markers has been only recently recognised.[3-5] Histological studies have shown various changes namely congestion, fibrosis, muscular deformities etc. It also showed immaturity of the ENS and alteration in density and distribution of ICC. These changes might be a cause of postoperative dysmotility responsible for soiling constipation, incontinence, etc. 

The ICC are considered to be the pacemaker cells of the gut which control and coordinates gut muscular activity. Recent report suggested that ICC express the tyrosine kinase receptor c-kit and that disruption of the c-kit signaling pathway inhibits differentiation of subpopulations of ICC. IHC staining using an anti c-kit antibody provides a sensitive technique for the identification of ICC which is also positive in mast cells. CD117 staining is typically cytoplasmic, with stronger accentuation along the cell membrane.[6,7] Calretinin is a vitamin D dependent calcium-binding protein used to identify ganglion cells. Both nuclear and cytoplasmic immune-reactivity is present in ganglion cells. S-100 a nerve sheath marker is used to identify the presence or absence of nerve hypertrophy in various cases of intestinal obstruction.[8,9] We studied histomorphological changes and various immunohistochemical (IHC) markers (calretinin, S-100, CD117) in intestinal wall specimens to assess neuronal dysfunction in a small cohort of ARM patients. 

## Material and methods:

Thirty patients with clinical diagnosis of ARM were included in the study. A representative biopsy was received in every patient. Specimen was fixed in 10% formalin fixative. The tissue was processed for paraffin embedding and wax block was prepared for routine histological technique and IHC stains. Histopathological diagnosis was established on routine H&E stain.[10] Following microscopic changes were observed on H&E: 

a) Presence or absence of ganglion cell; b) Hypertrophy of nerve fibers and trunk; c) Congestion and inflammatory infiltration in mucosa, submucosa and serosa; d) Hypertrophy of muscle layer (inner circular>outer longitudinal); e) Degeneration and fibrosis of muscles; and f) Muscle and serosal fibrosis.

Representative sections were also subjected to IHC staining11 for CD117, calretinin and S-100 protein. Paraffin sections (3-5 μm in thickness) on slides with suitable tissue adhesive were processed for deparaffinization and hydration. Endogenous peroxidase enzyme was inactivated by using 3% hydrogen peroxidase for 15-20 minutes. Antigen retrieval was done with microwave oven heating for 30 minutes with citrate. Sections then incubated with the monoclonal antibody (pre-diluted) (DAKO) overnight at 4°C. Then sections were rinsed with TBS solution followed by incubation with the secondary antibodies. The reaction was visualized using DAB (3,3′-*Diaminobenzidine*), and nuclei were counterstained with hematoxylin.

Positive and negative controls were run with each batch of IHC stain. Positive control for calretinin, S-100 and ICC were obtained from colonic tissue. Gastrointestinal stromal tumor tissue sections were used as positive control for CD117. Negative control was obtained by substituting the primary antibody with non-specific secondary antibody.

### Interpretation of results

For determination of density and distribution pattern of ICC, the cells were counted in 10 HPF (X400) in the muscular plane as well as myentric plexus separately. The density and distribution was compared to the control group. It was considered abnormal if relatively reduced number of positive cells or total loss of CD117 staining was observed. The percent of positive cells was semi-quantitatively estimated: 

a) Negative – none of the positive cells at 40X magnification; b) Weak positive (+ or grade 1) –visible cells only at 40X magnification or occasional weakly positive; c) Moderate positive (++ or grade 2) – easy distinguished cells at low magnification 10X; and d) Intense positive (+++ or grade 3) – cells intense positive at 10X magnification.[12] Ganglion cells showed nuclear and cytoplasmic positivity either within the submucosal nerve plexus or in the muscularis mucosae or in the lamina propria with calretinin. Schwann cells showed cytoplasmic positivity for S-100 and the ganglion cells were negatively stained. 

### Statistical Analysis 

A descriptive study was carried out for all the variables included in the study. Chi-square was used to compare the categorical values. P-value <0.05 was accepted as statistically significant. 

## Results

Age range in the patients of ARM was 1 day to 5 years with the mean age of 1.15 years. Maximum numbers of cases (n=15) were seen between age group of 1 month - 1 year (50%). Males (n=20) were predominantly affected over female (n=10) with a male: female ratio of 2:1.

All 30 cases with clinical diagnosis of ARM underwent surgical repair. At the time of restoration, full thickness biopsies were taken from sigmoid colon stoma. These biopsies were studied for histological changes using H&E stain. The most frequently observed histological finding in mucosa was inflammation and congestion in 87% and 67% of cases respectively. Disrupted muscularis mucosa was observed in 60%, eroded mucosa in 57%, and hemorrhage in 40% of cases. Submucosal inflammation and congestion were most common finding observed in submucosa in 87% and 80% cases respectively. Majority of cases of ARM included in our study showed chronic type inflammation. However grading of inflammation was not done in our study. Fibrosis was present in 67% (n=19) of cases; most of them (n=18) were associated with chronic inflammation. Other findings were haemorrhage widened submucosa in 47% cases each (Table 1). Fifty seven percent of cases showed thickening of muscularis propria. Of 30 cases of ARM, 5 cases showed presence of nerve bundle hypertrophy. Serositis was observed in 43% of the cases. (Figure 1) 

All the 30 cases of paediatric intestinal obstruction were examined for density and distribution of ICCs using CD117. In ARM cases, majority of them belong to grade 2+ category (n=17, 57%) followed by grade 1+ (n=8, 17%) (Figure 2) Seventy four percent of cases showed significant alteration in the density of ICC. Altered density and distribution was observed in ARM, which was statistically significant (p=0.02). (Table 2)

## Discussion

ARM are common congenital defects seen in paediatric surgery. Most of them cases are diagnosed just after birth; antenatal diagnosis is very rare. ARM present with wide range of malformations, that not only involves the anus and rectum, but it also involves urinary and genital tract. It has been observed that the pelvic floor and the smooth muscle of the terminal rectum in anorectal malformations remain mal-developed. There has been marked improvement in the diagnosis and management due to better understanding of anatomy embroyology and physiology of ARM and marked advancement in imaging studies. But still postoperative faecal and urinary incontinence is a big challenge. These complications are attributed to various histomorphological and neuronal dysfunction associated with ARM.[13]

In our study, we have observed vast number of histomorphological findings compatible with various studies and few findings contrary to previous studies. A number of new observations using IHC markers are also mentioned in our study with special emphasis on neuronal dysfunction. As stated by Tiwari *et al*., histopathological examination using H&E revealed numerous changes like inflammation, muscular hypertrophy, fibrosis and serositis etc.[5] (Table 3).

Agarwal *et al*.[3] reported hypertrophy of nerve bundles. Our study also showed nerve bundle hypertrophy (17%), which was confirmed using S-100 as immunohistochemical marker for nerve bundle. Dysganglionosis and aganglionosis have been frequently described in biopsies of the distal bowel in patients with ARM. Therefore, all the cases were examined for presence of ganglion cell on H&E stain as well as on IHC stain using calretinin. No abnormality was seen in ganglion cells in our study, which was in contrary to some of the studies published in the past. 

Recently, significant progress has been made in our understanding of ICC and their role in neonatal bowel obstruction and other congenital abnormalities of gastrointestinal tract. But limited data is available in respect to ARM. Variable results have been described in literature so far. Kenny *et al*.[14] observed alteration in density and distribution of ICC in most of their cases with 16% of cases showing complete absence of these cells. Similarly, we observed alteration in density and distribution of ICC was statistically significant (p=0.02). Contrary to this numerous studies like Jain *et al*.[15] and Strueker *et al*.[16] have shown complete absence, whereas Anatol *et al*.[17] stated no abnormality in Interstitial cells of Cajal. 

The underlying mechanism responsible for decrease in ICC in cases of ARM is not clear. Viral infection, unidentified toxin or metabolites or autoimmune mechanism may be involved. This finding has been proposed as an explanation for the persistence of constipation even after definite surgery in patients of ARM. Decrease density of ICC is also observed in cases of HD and allied disorder, two alternative interpretations have been proposed: either that the ENS is required for full differentiation of ICC or there is a common mechanism acting that affects both migration of neural crest derivatives and differentiation of ICC.[17] 

## Conclusion

Constipation is a common functional problem in patients operated for ARM, which has been assumed to be caused by associated motility dysfunction of recto-sigmoid. However. exact cause of this disorder is not known. Therefore, further studies including contractile function, electrophysiology, IHC and biochemical assay involving more number of cases are required for better interpretation and management of the problems.

## Footnotes

**Source of Support:** None

**Conflict of Interest:** None

## Figures and Tables

**Figure 1- a):**
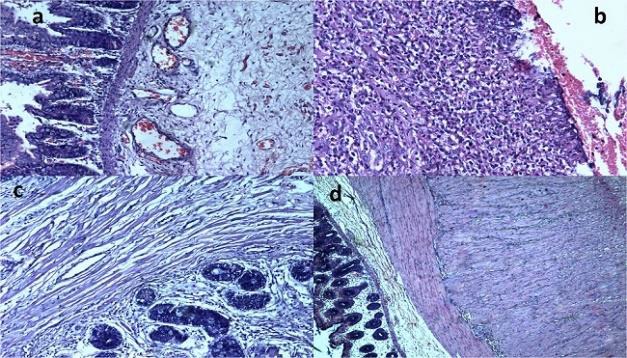
Anorectal malformation (ARM) showing congestion and oedema in mucosa and submucosa. (H&E, X200), b) Mucosal ero-sion and inflammation in ARM. (H&E, X200), c) Submucosal fibro-sis in ARM (H&E, X200), and d)Outer longitudinal layer of the muscularis propria showing muscle hypertrophy in ARM. (H&E, X100).

**Figure 1- a):**
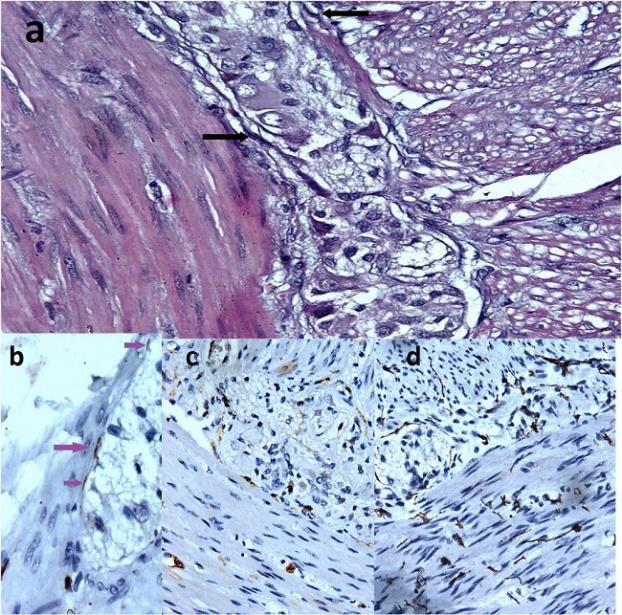
a) Interstitial cells of Cajal (ICCs) (arrow) around the myentric plexus (H&E, X400), b) Grade 1 immunostained intersti-tial cells of Cajal in Non-HD motility disorder – A few weakly posi-tive cells (arrow). (CD117 stain, X400), c) Grade 2 immunostained interstitial cells of Cajal in Non-HD motility disorder - easily distin-guished cells. (CD117, X200), and d) Grade 3 immunostained in-terstitial cells of Cajal in Non-HD motility disorder - intense posi-tive cells. (CD117, X200)

**Table 1:** Histological changes in mucosa and submucosa in cases of anorectal malformation

PARAMETERS	MUCOSA		SUBMUCOSA	
	P	A	P	A
Eroded mucosa	17	13	-	--
Widened submucosa	-	-	14	16
Congestion	20	10	24	06
Haemorrhage	12	18	14	16
Inflammation	26	04	26	04
Disrupted muscularis mucosa	18	12	-	-
Fibrosis	-	-	20	10

P: Present; A: Absent

**Table 2:** Distribution of cases according to grading of interstitial cells of Cajal (ICCS) using CD117 immunostain in cases of anorectal malformations

GRADING	No of cases	Percentage	P- value
**0**	-	-	0.020
**1+**	08	26%	
**2+**	17	57%	
**3+**	05	17%	
**Total cases**	30	100%	

Chi- Square test, degree of freedom =2

**Table 3:** Comparison of histological changes in bowel wall of ARM with other studies

Intestinal wall Histological changes		Tiwari et al^8^	Agarwal et al^9^	Present study
**Mucosa**	**Eroded mucosa**	**-**	**-**	**57%**
	Congestion	-	-	67%
	Haemorrhage	40%	-	40%
	Inflammation	60%	Most common finding	87%
	Disrupted muscularis mucosa	53.3%	-	60%
**Submucosa**	Widened submucosa	66.6%	-	47%
	Congestion	-	-	80%
	Haemorrhage	60%	-	47%
	Inflammation	80%	Most common finding	87%
	Fibrosis	-	-	67%
